# Enhanced Activity of Genes Associated With Photosynthesis, Phytohormone Metabolism and Cell Wall Synthesis Is Involved in Gibberellin-Mediated Sugarcane Internode Growth

**DOI:** 10.3389/fgene.2020.570094

**Published:** 2020-10-28

**Authors:** Rongfa Chen, Yegeng Fan, Haifeng Yan, Huiwen Zhou, Zhongfeng Zhou, Mengling Weng, Xing Huang, Prakash Lakshmanan, Yangrui Li, Lihang Qiu, Jianming Wu

**Affiliations:** ^1^Sugarcane Research Institute, Guangxi Academy of Agricultural Sciences/Sugarcane Research Center, Chinese Academy of Agricultural Sciences, Nanning, China; ^2^Key Laboratory of Sugarcane Biotechnology and Genetic Improvement (Guangxi), Ministry of Agriculture, and Guangxi Key Laboratory of Sugarcane Genetic Improvement, Nanning, China

**Keywords:** Transcriptome, co-expression network analysis, exogenous gibberellin, sugarcane, internode growth

## Abstract

Internode elongation is an important trait in sugarcane as it affects the sugarcane yield. Gibberellin (GA) is a key modulator of internode elongation in sugarcane. Understanding the gene expression features of GA-mediated internode elongation has both scientific and practical significance. This study aimed to examine the transcriptomic changes in the internode elongation of sugarcane following GA treatment. Eighteen cDNA libraries from the internode tissues on days of 0, 3, and 6 in control and GA treatment groups were sequenced and their gene expression were studied. RNA-seq analysis revealed 1,338,723,248 reads and 70,821 unigenes from elongating internodes of sugarcane. Comparative studies discovered a large number of transcripts that were differentially expressed in GA-treated samples compared to the control. Further analysis revealed that the differentially expressed genes were enriched in the metabolic process, one-carbon compound transport, and single-organism process. Kyoto Encyclopedia of Genes and Genomes pathway annotation showed significant enrichment in photosynthesis and plant hormone signal transduction, indicating its involvement in internode elongation. The function analysis suggested that metabolic pathways and biosynthesis of secondary metabolites, plant hormones, and cell wall components were enriched in the internodes of the GA-treated plants. The hub genes were identified, with the function of cellulose synthesis. The results of this study provide a global view of mRNA changes during sugarcane internode elongation and extend our knowledge of the GA-mediated cellular processes involved in sugarcane stem growth.

## Introduction

Sugarcane is a major agricultural crop in China, which as a country provides ∼80% of sugar produced globally (Chen et al., 2019), the second largest bioenergy crop in the world (Tomes et al., 2011). Sucrose is stored in the sugarcane stem and shoot height is an important determinant of sugar yield (Spaunhorst et al., 2019). Shoot length in graminaceous plants is largely determined by internode elongation, which is controlled the genetic make-up (Qi et al., 2011; Li et al., 2012), environmental factors (Mazzella et al., 2000; Peralta et al., 2002), and hormonal interactions (Buck-Sorlin et al., 2005; Liu et al., 2016). Plant growth regulators, such as auxin, gibberellins (GAs), ethylene, and abscisic acid (ABA) are the key regulators of internode elongation (Santner et al., 2009). Sugarcane stem growth is highly susceptible to abiotic stresses, and the majority of commercial sugarcane crop is subject to some level of abiotic stresses during the crop cycle (Lakshmanan and Robinson, 2014). Strategies to promote internode elongation are important for improving the sugarcane yield.

Gibberellins are widely used in horticulture and agriculture to increase crop productivity and performance (Basra, 2000). They play important roles in regulating various plant developmental processes, including seed germination, stem and hypocotyl elongation, leaf morphogenesis, floral development, and fruit maturation (Claeys et al., 2014). Gibberellin was first found in rice plants infected with the pathogenic fungus *Gibberella fujikuroi* (Binenbaum et al., 2018). To date, more than 130 GAs were identified in plants, fungi, and bacteria. However only a few of them, such as GA1, GA3, GA4, and GA7, function as bioactive hormones (Binenbaum et al., 2018). Gibberellins are produced primarily by the methylerythritol phosphate pathway. It has been generally assumed that the trans-geranylgeranyl diphosphate (GGPP) is first converted to ent-kaurene, then to GA12 by ent-kaurene oxidase, and ent-kaurenoic acid oxidase. The GA12 is converted into GA53, which finally produces the biologically active forms that are catalyzed by the oxoglutarate-dependent dioxygenases GA3ox (GA 3-oxidase; Amorim et al., 2017). In the growth hormone of plants, brassinosteroid (BR) and photoperiod can regulate the synthesis of the GA and its biological activity.

Gibberellins are known for their role in stem elongation by promoting both cell elongation and cell division (Rademacher, 2000). Gibberellin regulates plant growth by reducing the activity of DELLA nuclear repressor proteins and interacting with other hormone signaling pathways, such as auxin and BR (Nemhauser et al., 2006; Chaiwanon et al., 2016). The over-expression of genes encoding GA biosynthesis enzymes can increase the GA content, resulting in significant increases in shoot growth and xylem lignification in tobacco (Israelsson et al., 2003). In contrast, suppression of the expression of *GA20ox1*, the key GA biosynthesis gene has reduced rice plant elongation growth (Oikawa et al., 2004). Studies have shown that GA deficiency results in severe dwarfism and abnormal stem elongation of sorghum (Ordonio et al., 2014), and sugarcane (Garcia Tavares et al., 2018). These results indicate the important role of GA in controlling stem development in plants including sugarcane.

Modification of endogenous GAs increases plant vegetative biomass, alters shoot architecture, and controls fruit and seed development (Basra, 2000; Wang Z. et al., 2012; Cheng et al., 2013; Zhang et al., 2018). Modification of endogenous GAs in sugarcane by either altering regulatory genes involved in its metabolism (Pribil et al., 2007), changing the activity of GA action modulator DELLA protein (Garcia Tavares et al., 2018), or by external application of GAs significantly increased stem growth and biomass (Moore and Buren, 1978).

Our earlier studies indicated that miRNA–mRNA pairs are involved in zeatin biosynthesis, nitrogen metabolism, and plant hormone signal transduction pathways, and are strongly associated with internode elongation and development in sugarcane (Qiu et al., 2019a). Moreover, GA content in leaves significantly increased after GA spraying (Qiu et al., 2019b). We also found that the expression level of GA20 ox1 and gibberellin insensitive dwarf 1 (GID1) was increased on days 3 and 6, and then decreased on days 12 and 24 after GA3 treatment in sugarcane (Qiu et al., 2019b). These results indicated that GA stimulated the sugarcane internode elongation by increasing its endogenous levels and possibly regulating the expression of a multitude of growth and developmental genes. The molecular mechanism underpinning GA-mediated sugarcane stem growth and sugar accumulation remains unclear. Therefore, in the present study, we conducted a detailed analysis of gene expression profiles during GA-induced internode elongation in sugarcane using Illumina Hiseq 4000 platform. The results presented here provide new insights on stem growth and sugar accumulation, the two most economically important traits in sugarcane.

## Materials and Methods

### Preparation of Test Materials

The experiment materials used in this study included a sugarcane variety GT42 bred by the Sugarcane Research Institute (SRI), Guangxi Academy of Agricultural Sciences, Nanning, China. The plant material was sourced from an SRI Experimental Farm at Nanning, China, where the sugarcane variety was grown following local crop management practices. As reported earlier (Wang L.W. et al., 2015), we selected 10-month mature cane stalks with buds in the middle, and then cut into setts with single-bud, and then incubated it at a temperature of 52°C for 30 min to remove the pathogenic bacteria. Subsequently, the setts were transferred into a moist sandbox to keep germination in an artificial climate box (Essenscien, United States). The conditions were as follows: temperature: 28 ± 0.1°C, humidity: 75 ± 1.5% RH, photoperiod: 100% full light (light intensity 25000 LX), 12 h light and 12 h dark. When buds grew into 2-leaf stage seedlings, the morphologically uniform plants were transferred to plastic pots (35 cm in inner diameter and 50 cm in height), each carrying two plants. Five days after transplantation, they were randomly divided into two replicates, each with 45 pots. When the sugarcane seedlings grew to the pre-elongation stage with 9–10 leaves (the early elongation stage), they were sprayed with gibberellin solution (GA3; concentration 200 mg/L) until drops flowed down from the leaves. The control group sugarcane seedlings were similarly sprayed with water. The transplanted sugarcane pots were placed in a greenhouse and put in 18 rows with a spacing of 1.2 m, and five sugarcanes were placed in each row with a spacing of 20 cm. Every sixth row was divided into one repeating block, the first three columns were the control group and the last three columns were the GA group. From each treatment group, the third internodes from the shoot top were collected on days 0, 3, and 6 after spraying. The samples of internode tissues after 0, 3, and 6 days in the control group were named C1, C2, and C3 respectively, and in the GA treated group were named as G1, G2, and G3 respectively. They were immediately frozen in liquid nitrogen and stored at − 80°C until used. Each biological replicate had five internodes, and three biological replicates were included for each time point for both treatments.

### Measurement of Plant Height

Five sugarcane plants from each treatment group were selected randomly to measure stalk height (from the pot soil surface to the dewlap of the youngest fully expanded leaf) and the length of the internode on the 0, 3, and 6 days after spraying. The whole plant height and the first seven internode lengths from the shoot top of 10 matured plants were measured.

### Construction of RNA Sequencing Libraries

The total RNA was extracted by using RNA Trizol (Invitrogen, Carlsbad, CA, United States) following the manufacturer’s instructions. The integrity and quantity of the extracted RNA were determined by an Agilent 2100 bioanalyzer (Agilent, Santa Clara, CA, United States). Six RNA libraries were constructed in this study (three treated groups and three control groups). From the total RNA samples, the mRNA was isolated by magnetic beads with oligo (DT). The enriched mRNA was fragmented into short fragments using a fragmentation buffer. Then, using the short mRNA fragment as the temple, the first strand of cDNA was transcribed by six-base random primers, followed by synthesis of the second strand cDNA using the buffer, dNTPs, RNase H, and DNA polymerase I. Subsequently, the cDNA fragments were purified by Qiaquick PCR extraction kit (Qiagen, Hilden, Germany), end repaired, poly(A) added, and ligated to Illumina sequencing adapters. Finally, the ligation products were recovered by agarose gel electrophoresis and then processed the PCR amplification to complete the library preparation. The constructed libraries were sequenced using Illumina HiSeqTM 4000 by Gene *Denovo* Biotechnology Co. (Guangzhou, China).

### Mapping of Clean Reads, Analysis of Differentially Expressed Genes

The raw data obtained from the sequencing machines contained adapters or low-quality reads. They were filtered to remove the adapters and reads with an unknown nucleotides (N) ratio of >10%. Subsequently, low-quality reads with 40% bases and Q-value ≤ 20 were removed. The high-quality clean reads without the rRNA were used for further analysis. Then the clean reads were mapped with the Genomic data from NCBI Genome Database (Bioproject Accession Number: PRJNA431722) using short reads alignment tool bowtie2 by default parameters and obtained mapping ratio. The gene abundances were calculated and normalized using the method of RPKM (Reads Per kb per Million reads), which can eliminate the influence of different gene length and sequencing data on the calculation of gene expression. Therefore, the calculated gene expression can be directly compared to the difference in gene expression among samples. Principal component analysis (PCA) was performed with R package models (http://www.r-project.org/). To identify differentially expressed genes between different groups, the edgeR package (http://www.r-project.org/) was used. We identified genes with a fold change | log2FC| > 1 and a false discovery rate (FDR) < 0.05 in comparison as significant differentially expressed genes (DEGs).

### Gene Ontology and Kyoto Encyclopedia of Genes and Genomes Pathway Analysis

To identify the biological process and signaling pathways of the genes, we performed an analysis of Gene Ontology (GO) and pathway enrichment using the Kyoto Encyclopedia of Genes and Genomes (KEGG) with the DAVID online tools (http://david.ncifcrf.gov/). The GO items with FDR ≤ 0.001 and | log2Ratio| ≥ 1 were recognized as the enriched GO items. Pathways with FDR ≤ 0.01 were defined as significantly enriched pathways.

### Weighted Gene Co-expression Network Analysis

To study the gene function associated with sugarcane growth, the DEGs between GA-treated group and the control group were selected and analyzed using weighted co-expression network analysis by using the R package weighted gene co-expression network analysis (WGCNA). Firstly, the genes that showed a 25% variation were selected based on variance (Standard Deviation/Mean) across samples and used for network construction. We established a matrix of correlation between all the DEGs by the expression value, and a weighted adjacency matrix was created with the soft threshold power (β) set at 8 to analyze scale-free topology. Then the gene module was identified by the parameters with a power of 8, min ModuleSize of 30, and branch merge cut height of 0.25. The correlation of the eigengene module was determined in the study. The correlations of the groups and modules were assigned correlation coefficients from -1 to 1. The significant modules correlated with GA-treated were analyzed in the following study. The GO and KEGG enrichment analyses were performed. The correlation of key hub genes in the modules was analyzed by Cytoscape v3.7.1.

### Real-Time Quantitative PCR Analysis of Genes

The internode tissues of GA-treated and control plants were collected at different time points as described in section “Preparation of Test Materials.” The total RNA of tissues was extracted by using RNA Trizol (Invitrogen, Carlsbad, CA, United States). The primers of the tested genes were designed by Primer Premier 5.0 (Applied Biosystems, Waltham, MA, United States) (Supplementary Material 1). The cDNA was synthesized using PrimeScript RT Reagent Kit with gDNA Eraser, diluted tenfold, and used as the template for Real-time Quantitative PCR (qPCR). The PCR reaction program was 95°C for 5 min for initial denaturation, followed by 45 amplification cycles at 95°C for 10 s and 60°C for 20 s using the AnalytikJena qTOWERE2.2 fluorescence quantitative PCR instrument (Germany). At end of the amplification, a melting curve analysis was conducted to detect the specificity of the reaction. All the reactions were performed with five biological replicates. The gene relative expression level was calculated using the 2^–Δ^ ^Δ^ ^CT^ method (Livak and Schmittgen, 2001).

### Data Statistical Analysis

Results of growth rate, sugarcane height, internode length, and qPCR are presented as the means ± standard deviations (SD). The obtained data were analyzed statistically to determine the degree of significance between control groups and treatment groups using a one-way analysis of variance (ANOVA) on SPSS statistical software package (SPSS for Windows, version 19.0, SPSS, Chicago, IL, United States). Differences between the mean values were considered to be significant at *p* < 0.05.

## Results

### Gibberellin Application Greatly Increased Sugarcane Shoot Growth Rate

**FIGURE 1:**
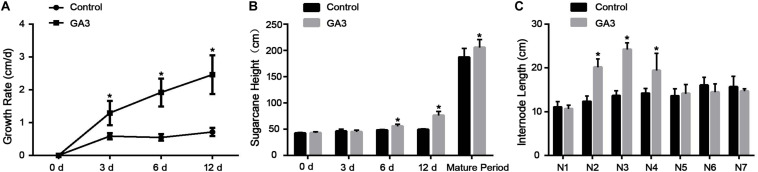
Effects of GA3 on sugarcane phenotype on different days after treatment. **(A)** The growth rate of sugarcane on different days after GA3 treatment (*n* = 4). **(B)** The height of mature sugarcane with or without GA3 treatment (*n* = 4; mature period, *n* = 10). **(C)** The internode length of sugarcane in mature sugarcane after GA3 treatment. The asterisk indicates significant differences between control and GA3 treatment groups (*n* = 5, *P* < 0.05).

Sugarcane plants treated with GA showed a significantly higher growth rate than the control plants on 3, 6, and 12 days after treatment (Figure 1A). And, the same plants were significantly taller than the control group at maturity (Figure 1B). Measurement of internode length of mature sugarcane plants showed that the second, third and fourth (N2, N3, and N4 from the shoot top) internodes were significantly longer than that of the control group. No difference was observed in the N1, N5, N6, and N7 internodes (Figure 1C).

### Differentially Expressed Genes in Gibberellin Treated Internodes

To understand the growth effect and the molecular changes caused by GA treatment in sugarcane, 18 cDNA libraries constructed from both GA-treated and control plant internodes were sequenced and the data were analyzed. A total of 1,362,424,878 reads were obtained from all the libraries. The sequences are available on the NCBI bioproject (PRJNA633918). There were 1,338,723,248 clean reads identified after filtering (Supplementary Material 2).

**FIGURE 2:**
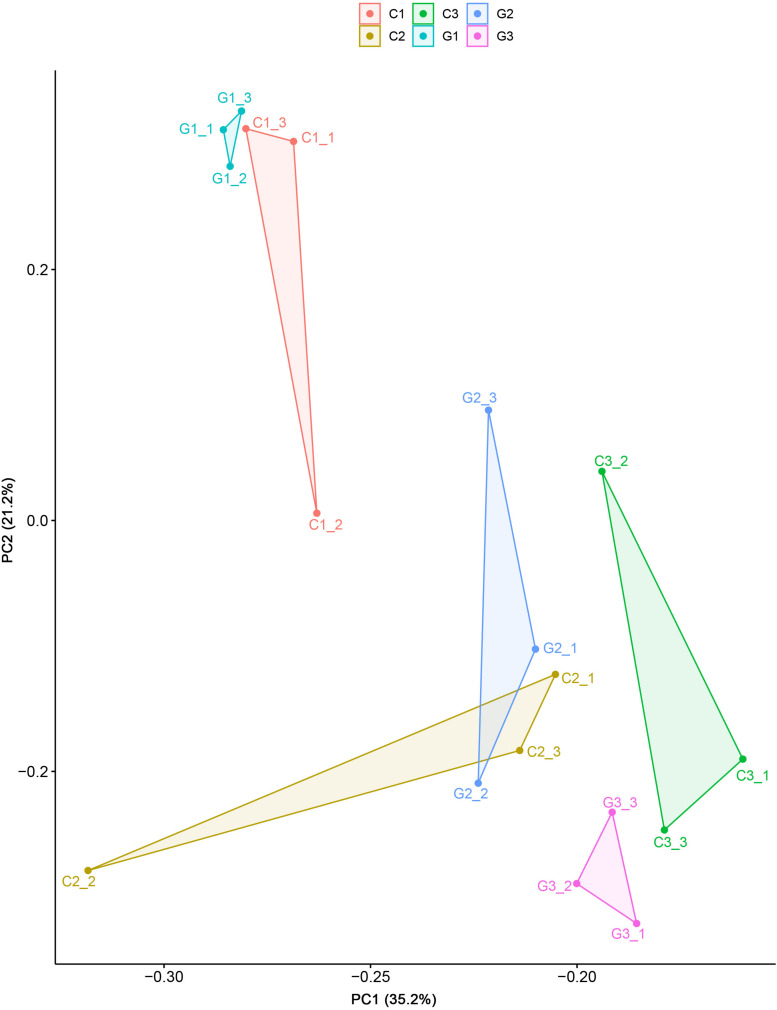
Principle component analyses of the 18 transcriptomes from the elongation tissues on different days in the control and GA3 treatment groups based on the variation of DEG expression.

All the clean reads were mapped to the reference transcriptome sequence and generated 70,821 unigenes. The gene expression in the present study was shown in Supplementary Material 3. For the next comparison, we applied the PCA using all gene expression data of all the groups and observed that the first two principal components separated the different treatment groups, indicating strong transcriptional differentiation among the studied groups (Figure 2).

**FIGURE 3:**
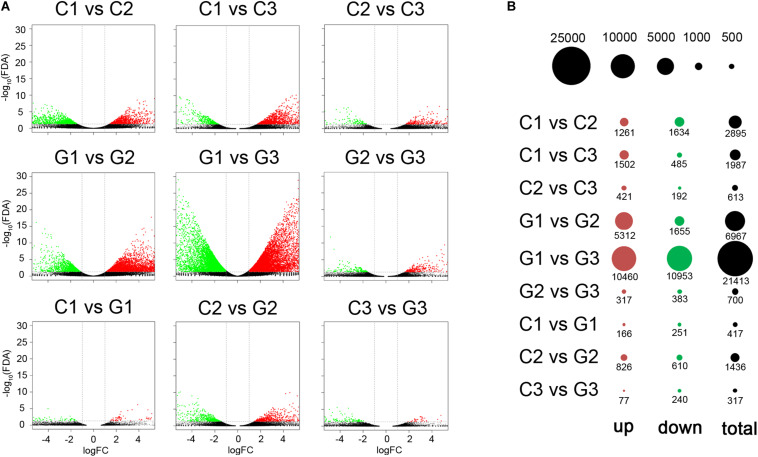
DEGs determined by pairwise comparison on different days in control and GA3 treatment groups. **(A)** Volcano plots showing the differentially expressed genes in the comparisons. Red and green dots represent the up- and down-regulated genes, respectively. **(B)** Bubble plot showing the distribution of the differentially expressed genes for comparisons. The red and green circles show up- and downregulated genes. The size of the circle was drawn to show the counts of differentially expressed genes.

The edgeR package was used to identify the differentially expressed genes between GA-treated group and the control group (Supplementary Material 4). In comparison between C1 and C2, C1 and C3, C2 and C3, 2,895, 1,987, and 613 differentially expressed genes were identified, respectively (Figure 3A). When compared to G1, there were more differentially expressed genes in G2 and G3, with 6967 (5312 upregulated and 1655 downregulated genes) and 21,413 genes (10,460 upregulated and 10,953 downregulated genes), respectively. While only 700 genes were differentially expressed between G2 and G3. Compared to the C1, 417 differentially expressed genes (166 upregulated and 251 downregulated genes) were found in the G1 group. A total of 1436 differentially expressed genes (826 upregulated and 810 downregulated genes) were found when comparing the C2 and G2 groups. Between the C3 and G3 groups, 77 genes were up-regulated and 240 genes were down-regulated (Figure 3B).

### Functional Annotation of the Differentially Expressed Genes

**FIGURE 4:**
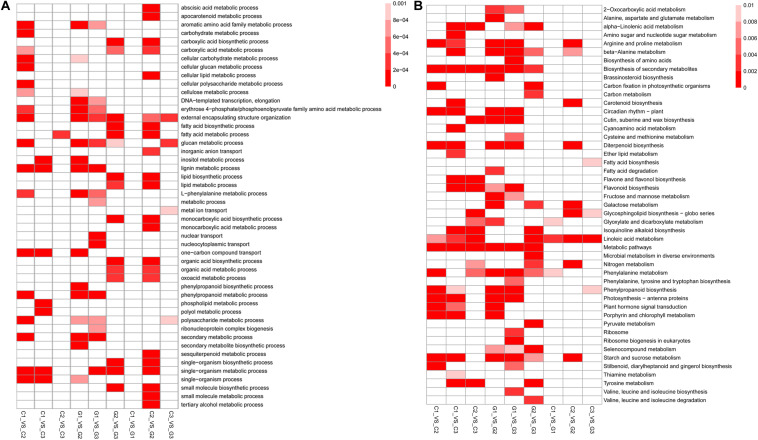
Functional analysis of the DEGs identified between different treatment groups. **(A)** Heatmap showing that the GO enrichment analysis of biological processes between different treatment groups (*P* < 0.005). **(B)** Heatmap indicating the KEGG pathway enrichment analysis of DEGs identified between different treatment groups (*P* < 0.05). The color in the boxes shows the correlation according to the color legend.

To reveal the function of the differentially expressed genes among the tested groups, we conducted an enrichment analysis of the biological process in GO and KEGG pathways. It was found that the comparison of C1 and G2 had the most GO terms with 22 enriched GO terms, while the C1 and G1 had the least with no enriched GO terms (q-value < 0.001). Between C1 and C2, there were 18 Go terms enriched. Between the C1 and C3, there were eight enriched GO terms, including various metabolic processes, one-carbon compound transport, and single-organism processes. Only 1 GO term, the fatty acid metabolic process was discovered in the comparison C2 vs C3 (Figure 4A and Supplementary Material 5). Most of the GO terms were involved in the metabolic processes associated with glucan, polysaccharide, other carbohydrates, and phenylpropanoid compounds. The results indicate that GA may accelerate metabolism and promote stem and hypocotyl elongation.

The enrichment analysis of KEGG pathways showed that several metabolic pathways were enriched by pairwise comparisons. Only 4 KEGG pathways were identified as enrichment pathways comparison between C3 and G3 including fatty acid biosynthesis, glycosphingolipid biosynthesis, phenylpropanoid biosynthesis, and linoleic acid metabolism. The comparison between G1 and G3 had 23 enriched KEGG pathways which were the largest in the comparisons. The KEGG pathways contained lipid, carbon, and nitrogen metabolisms and cutin, suberine, and wax biosynthesis. Other comparisons also demonstrated that the enriched KEGG pathways were associated with plant growth (Figure 4B and Supplementary Material 6).

### Weighted Gene Co-expression Networks Analysis

**FIGURE 5:**
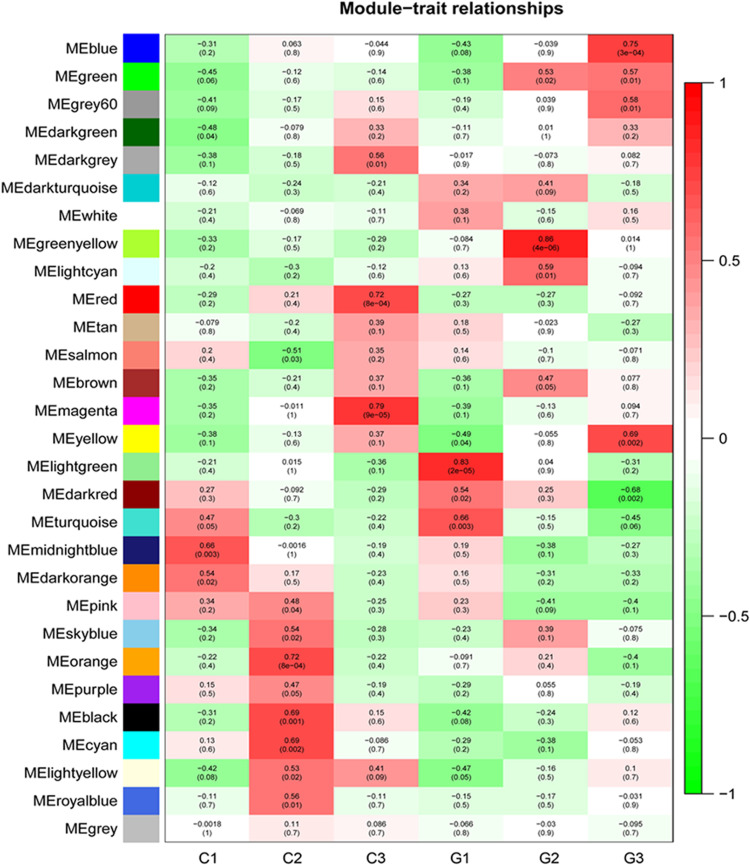
WGCNA analysis of transcriptome during internode elongation as affected by GA treatment. Module-trait relationship heatmap for different traits and gene modules. The yellow-green modules are positively related to G2. The blue modules are positively related to G3, while dark red is negatively associated with G3. The values in the figure indicate the correlation coefficient between modules and traits. Values in brackets are the *P*-values for the association test.

**FIGURE 6:**
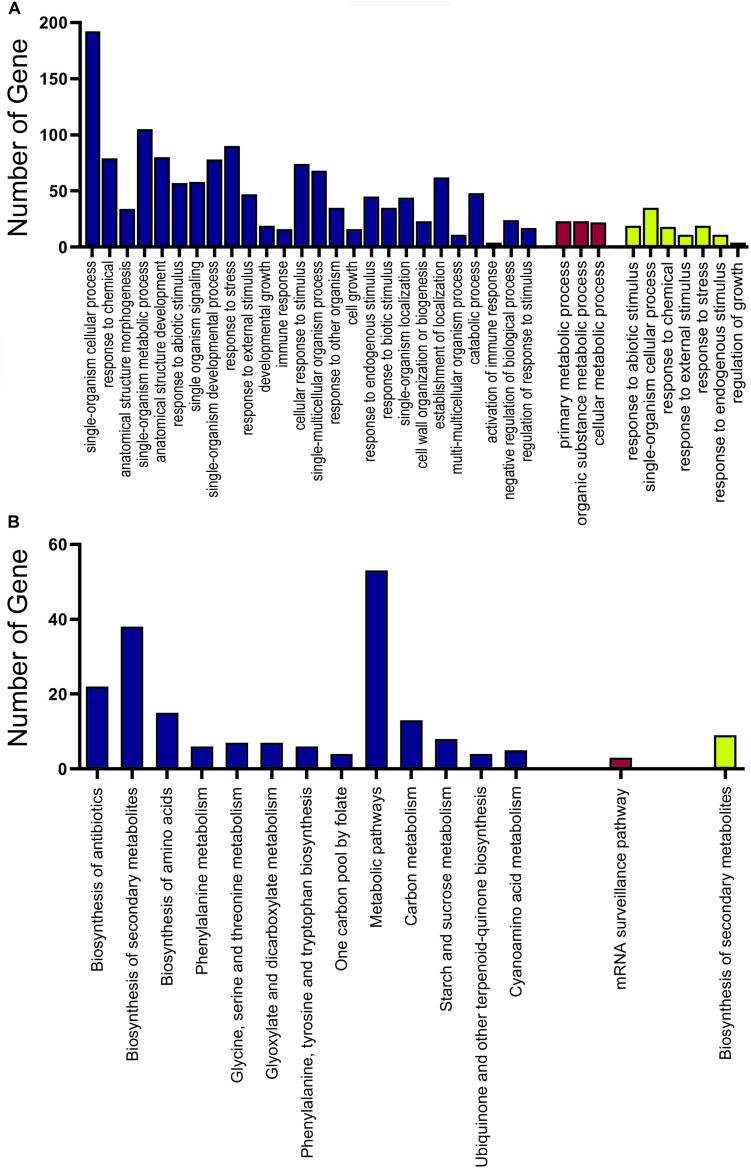
Functional analysis of the genes in yellow-green, blue, and dark red modules. **(A)** GO enrichment analysis of gene in the yellow-green, blue, and dark red modules. **(B)** KEGG pathway enrichment analysis of gene in the yellow-green, blue, and dark red modules. The yellow-green indicated the GO and KEGG pathway enrichment analysis of the yellow-green module. The blue indicated the GO and KEGG pathway enrichment analysis of the blue module. The dark red indicated the GO and KEGG pathway enrichment analysis of the dark red module.

Based on the variance (Standard Deviation/Mean) across samples, the top 15,896 genes with a significant variation for activity were selected for the network construction. Twenty-eight modules were detected by the WGCNA (Supplementary Figure 1). The turquoise module was the largest module with 6881 genes, while the white module only had 68 genes, the minimum module (Supplementary Material 7). Among these modules, three of them were significantly enriched in GA-treated samples (Supplementary Material 8). Genes in module “green-yellow” were positively correlated with G2 (*p* = 0.86). The module of blue was positively correlated with G3 (*p* = 0.75), while the dark red module was negatively correlated with G3 (*p* = -0.68) (Figure 5). The genes in green-yellow module were enriched in response to a stimulus, such as abiotic and stress, endogenous signals, single-organism cellular processes, and regulation of growth. As shown in Figure 6, the green-yellow module was showed activated biosynthesis of secondary metabolites. The GO terms including cellular processes, metabolic processes, structure development, and stress response were mainly enriched in the blue module. Module dark red was enriched in metabolic processes (Figure 6A). In green-yellow, the biosynthesis of secondary metabolites was identified. The biological activities enriched in the blue module were the metabolic pathways, particularly the biosynthesis of secondary metabolites, antibiotics, and amino acids, and other related compounds. Only the mRNA surveillance pathway was deleted in the dark red module (Figure 6B).

The hub genes in the modules of green-yellow, blue, and dark red were analyzed by Cytoscape. The genes of LUX, AFP-D1, and CLPB1 were the most connected hub in the module of green-yellow (Supplementary Figure 2A). In the blue module, gene Gt43C, CESA9, and GLU10 were in the center of the network, which connected with 1150 transcripts in all (Supplementary Figure 2B). TIR1, CKL, and wdr4 were the hub genes of the dark red module. In the dark red module network, 77 transcripts were linked to the three genes (Supplementary Figure 2C).

### Verification of Gene Expression Levels by qRT-PCR

**FIGURE 7:**
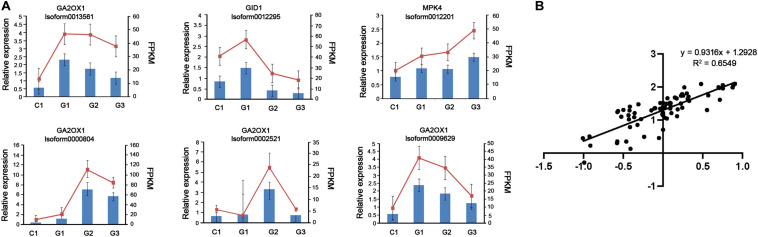
RNA-seq data validation by qRT-PCR. **(A)** The histograms show the qPCR results of 6 unigenes involved in GA biosynthesis on different days after GA treatment. The red line charts show the FPKM values of these unigenes, and blue bars show the qPCR results (*n* = 3). The left Y-axis indicates the relative expression levels calculated by qPCR and the right Y-axis indicates the FPKM values of RNA-seq data. **(B)** Comparison between the log2 fold-changes calculated based on the RNA-seq and qRT-PCR data.

To confirm the accuracy and repeatability of the RNA-seq data, we randomly selected six DEGs involved in GA signaling for qRT-PCR analysis using mRNA extracted from C1, G1, G2, and G3 stage. The comparative analysis of all the selected genes showed that qRT-PCR analysis had similar expression patterns to the RNA-seq data, suggesting the reliability of RNA-seq results. The log2 fold-changes were based on the qRT-PCR and RNA-seq data and were highly correlated [y = 0.9316x + 1.2928; R^2^ = 0.6549] (Figure 7). In particular, the expression levels of Isoform0012295 (GID1), Isoform 0013561 (GA2ox1), and Isoform 0009629 (GA2ox1) were downregulated on days 3 and 6 after GA treatment.

## Discussion

Gibberellin is commonly used in agriculture, horticulture, and floriculture globally to improve crop yield and product quality (Phillips, 2018). In the present study, we used transcriptomic approaches to study shoot growth and the activity of sugarcane genes as affected by GA treatment.

It was established that the GA is involved in internode elongation in plants (Hedden and Sponsel, 2015). For example, in rice seedlings showed shoot elongation 24-h after GA treatments (Qi et al., 2011). Our results showed that on days 6 and days 12 following GA treatment, the height of GA-treated sugarcane was significantly higher than that of control plants. This was consistent with our previous studies (Qiu et al., 2019b). In addition, the average daily growth rate of sugarcane was significantly higher than the control plants, with approximately 2 times on day 3, and 3.5 times on both days 6 and 12. In the mature plants, the height of GA-treated plants was significantly higher than the control. The internode length of sugarcane, particularly N2, N3, and N4 were longer than the corresponding ones in the control plant, which indicate that the GA mainly promoted the elongation growth when the internode is at the peak elongation stage.

In the present study, 18 internode tissue samples collected from GA-treated and control plants on days 0, 3, and 6 after GA treatment were used to analyze GA-induced transcriptome changes during internode elongation. The *de novo* generated 70,821 unigenes. A large number of DEGs were differentially expressed during internode after GA treatment through RNA-seq analysis. The RNA-seq results suggested that the number of differentially expressed genes in the C1 vs. C2, C1 vs. C3, and C2 vs. C3 comparisons were 2895, 1987, and 613, respectively. While 6967 and 21413 genes were found to expresses differences in G2 and G3 respectively when compared to the G1. We also mentioned that the DEGs between the control and GA treated groups were smaller than that when G1 was compared with G2/G3. Surprisingly, C2 vs G2 and C3 vs G3 comparisons had fewer DEGs than that in the G1 vs G2 and C3 vs G3 comparisons. The gene expression changes mainly occur in the different treated stages of GA. In the same stage, the gene expression changes are smaller between GA and control groups. We proposed that the GA treatment predominantly affected the internode elongation in the long term. Thus, in terms of gene expression, the GA had a greater effect on the internode gene transcription activity.

By analyzing GO enrichment, we identified DEGs that participated in the biological process category. When compared with G1, the major representation of genes in G2 and G3 stage were found to be under the category of metabolic processes, such as lignin, cellulose, cellular carbohydrate, and glucan metabolism, indicating the function of these genes in internode elongation. Gene Ontology analysis of moso bamboo, which was treated with exogenous GAs, showed that the DEGs are mostly associated with photosynthesis, lignin metabolism, response to chemical stimulus, and others (Zhang et al., 2018), similar to our results. Lignin is the key structural component of xylem vessels and it provides mechanical strength to plants (Rogers and Campbell, 2004). Cinnamoyl-CoA reductase (CCR), LAC4, and cinnamyl alcohol dehydrase (CAD) are the key lignin biosynthesis related genes (Liu et al., 2018). In GA3 treated moso bamboo, CCR, and LAC4 gene expression was higher (Zhang et al., 2018). In tobacco, the downregulation of CCR significantly reduced lignin contents, and the rate of plant development (Chabannes et al., 2001). In rice, mutations of gene CAD have an influence on lignin deposition, which causes semi-dwarfism, lower grain yields, and reduced culm stiffness (Hirano et al., 2012). We also found that the “Inositol metabolic process” was enriched in the DEGs when compared to G1 vs G2 and G1 vs G3. Inositol metabolism is crucial for xylan and pectin production, which are two important structural components of the cell wall (Stevenson et al., 2000). The significant changes in the activity of genes associated with xylan and the pectin biosynthetic pathways reflect the accelerated synthetic activity of the cell wall in GA-treated plants. Therefore, the effect of GA is regulated by multiple factors and our study shows that GA promotes growth through the activation of multiple genes associated with growth and development.

To understand the key metabolic pathways associated with GA-induced sugarcane internode elongation, the enriched KEGG pathways with the differentially expressed genes were identified. Photosynthesis, antenna proteins, plant hormone signal transduction, and cutin, suberine, and wax biosynthesis pathways were enriched with differentially expressed genes in G2 and G3 compared to G1. The differentially expressed genes in photosynthesis, for example in antenna proteins pathways, included CABs, LHCB, and RCABP89. Previous studies have reported a negative relationship between GA and photosynthesis (Stavang et al., 2009; Gururani et al., 2015). In broad bean, soybean, and populous, GA promoted photosynthesis (Yuan and Xu, 2001; Tian et al., 2016), while in Plantago, the external application of GA, decreased the rate of photosynthesis (Dijkstra et al., 1990). In our study, the gene expression of CAB1, CAB7, and LHCB5, was higher in G2 and G3 than in G1, which indicated that GA treatment may have a positive effect on sugarcane photosynthesis including in CAB, a key chlorophyll biosynthesis gene (Chen et al., 2019). The increased expression of the genes in these pathways might be related to high growth activities of GA3-treated sugarcane, which enhances photosynthesis. It is established that the stem elongation is normally associated with the level of hormones and interaction among them. The GA, indole acetic acid (IAA), and ABA are important hormones in the stem elongation process (Tomes et al., 2011). Indole acetic acid is a growth hormone that promotes bud outgrowth and branching in plants (Lakshmanan and Robinson, 2014). In San Pedro-type fig (*Ficus carica*), IAA content was significantly decreased in the GA-treated flowers, while the ABA level was increased compared to the control (Garcia Tavares et al., 2018). These results collectively suggest that exogenous GA treatment affects photosynthesis and endogenous hormone content, modulating the plant development, as seen in sugarcane in this study. Furthermore, it is likely that GA regulates sugarcane growth by interacting with other hormones as well.

Weighted gene co-expression network analysis is a powerful tool in analyzing the relationship of the gene networks and phenotypes, as it provides insights into the gene function and the control mechanisms of complex traits (Langfelder and Horvath, 2008). By using WGCNA, the glucosinolate metabolism regulation genes were identified in Chinese kale (Moore and Buren, 1978). Similarly, it was successfully applied to identify key metabolic genes of aporphine alkaloid in *Nelumbo nucifera* (Bolduc and Hake, 2009) and flavonoid biosynthesis in *Ginkgo biloba* (Ye et al., 2019). Although the transcription genes overlapped in different development stages of sugarcane, some genes are specifically transcribed in a specific stage. It distinguishes the genes involved in internode elongation after GA treatment.

Our WGCNA study identified 28 modules, and green-yellow, blue, and dark red modules showed significant correlations with GA treatment. The green-yellow module showed a positive relationship with the G2 stage. The blue module had a positive correlation with G3, while the dark red module had a negative correlation with G3. The green-yellow and blue modules showed the enrichment of functions involved in cell growth, regulation of growth, response to endogenous stimulus, and the metabolic process by GO annotation analysis. The GO enrichment in the dark red module identified major metabolic processes. In the KEGG pathway analysis, metabolic pathways and biosynthesis of secondary metabolites were enriched in green-yellow and blue modules, whereas, mRNA surveillance pathway was enriched in the dark red module. These results indicated that the expression of genes participating in the process of growth, metabolism, and response to endogenous stimulus were significantly affected by GA. Considering the positive relation of the modules with the GA treatment, we focused the analysis on the green-yellow and blue modules. Yellow-green modules contained the genes about the regulation of growth, response to endogenous stimulus, and single-organism cellular processes. The blue module was the larger module with the genes of response to stress, cell growth, response to stress, and other pathways. The GA treatment genes associated with growth regulation and phytohormone metabolism were stimulated, leading to the internode elongation of sugarcane.

The hub genes identified by the WGCNA are known for their roles in growth, with a combination of regulatory and biosynthetic functions. Cellulose synthase A catalytic subunit 9 (CESA9) is the hub gene in the blue modules, which is a part of the cellulose synthase. In higher plants, cellulose is synthesized at the plasma membrane by CESA enzymes, which is the principal component of plant cell walls (Langfelder and Horvath, 2008). All the CESA mutants showed the phenotypes with reduced cellulose and defective growth. The mutant of CESA9 rice is characterized by reduced cellulose, dwarfism and they have brittle culms that are easily broken (Wang D. et al., 2012). Furthermore, the overexpression of CESA genes could affect plant growth in transgenic plants (Li et al., 2017). These results indicated that CESA9 plays an important role in cell wall biosynthesis and plant growth. In our study, the CESA9 gene acts with other genes to accelerate cell wall synthesis and growth. Caseinlytic proteinase B1 (CLPB1) is a heat shock protein to modulate plant in the stress response at different developmental stages. It is usually clubbed with heat shock protein 100 (Hsp100) to form the ClpB/Hsp100 proteins for its function. The ClpB/Hsp100 mutant of *Arabidopsis*, maize, and rice show extreme sensitivity to heat stress (Lee et al., 2007). The over-expression of ClpB/Hsp100 in rice and Arabidopsis results in the enhancement of thermotolerance (Katiyar-Agarwal et al., 2001). With GA treatment, the expression of CLPB1 is increased, which indicating GA could enhance the heat resistance of the sugarcane.

The synthesis of GA is affected by many factors, and the exogenous supply of phytohormone can be used to regulate GA synthesis via feedback mechanisms. Gibberellin 2 Oxidase (GA2ox1) and gibberellin insensitive dwarf 1 (GID1) are the major genes of regulating the GA biosynthesis, and their expression levels are related to the changes in the endogenous GA level. In higher plants, the content of active GAs is regulated by the balance between their rates of biosynthesis and deactivation. GA2ox1 is the key enzyme to reduce the content of bioactive GA in culm, especially in the basal internodes (Bolduc and Hake, 2009). Studies have shown that overexpression of the GA2ox gene led to the enhancement of GA inactivation and induce dwarfism in *Solanum*, *Nicotiana tabacum*, *Oryza sativa*, *Arabidopsis thaliana*, and *Populus* (Zhou and Underhill, 2016). Gibberellin insensitive dwarf 1 is the key mediator of the GA response pathway. The expression of the GID1 gene in cotton, rice, and potato would be downregulated by the exogenous GA (Mauriat and Moritz, 2009). In our study, the expression levels of GA2ox1 and GID1 were both downregulated on days 3 and 6 after GA treatment. These results suggested that exogenous application of GA reduced the expression of GA2ox1, resulting in the accumulation of GA. The reduction in the expression of GID1 would act as negative feedback to GA activity.

## Conclusion

In summary, this comprehensive transcriptome analysis of the effects of exogenous application of GA on internode elongation of sugarcane identified the gene network associated with this biological process. The results suggest that genes involved in metabolic processes, photosynthesis, and plant hormone signal transduction pathways, play an important role in GA-induced growth in sugarcane. These findings provide a molecular platform for future studies on the molecular regulation of internode elongation in sugarcane.

## Data Availability Statement

The datasets presented in this study can be found in online repositories. The names of the repository/repositories and accession number(s) can be found below: https://www.ncbi.nlm.nih.gov/, PRJNA633918.

## Author Contributions

RC and YF performed the experiments. HY and HZ collected materials. ZZ, MW, and XH carried out the bioinformatic analysis and drew the figures. PL and YL wrote the manuscript. LQ and JW designed the study and guided the research study. All authors read and approved the final manuscript.

## Conflict of Interest

The authors declare that the research was conducted in the absence of any commercial or financial relationships that could be construed as a potential conflict of interest.

## Supplementary Material

Supplementary Figure 1 Cluster dendrogram of the clustering of dissimilarity using a consensus topological overlap. Each module color represents one module. A total of 29 modules were identified.

Supplementary Figure 2 Identification of hub gene in yellowgreen, blue and darkred modules by Cytoscape. **(A)** The hub gene in yellowgreen module. **(B)** The hub gene in blue module. **(C)** The hub gene in darkred module.
