# Supplementary figures and images for: Enhanced Activity of Genes Associated With Photosynthesis, Phytohormone Metabolism and Cell Wall Synthesis Is Involved in Gibberellin-Mediated Sugarcane Internode Growth
Source: Front Genet. 2020 Oct 28;11:570094. doi: 10.3389/fgene.2020.570094 (PMC7655795; doi:10.3389/fgene.2020.570094)

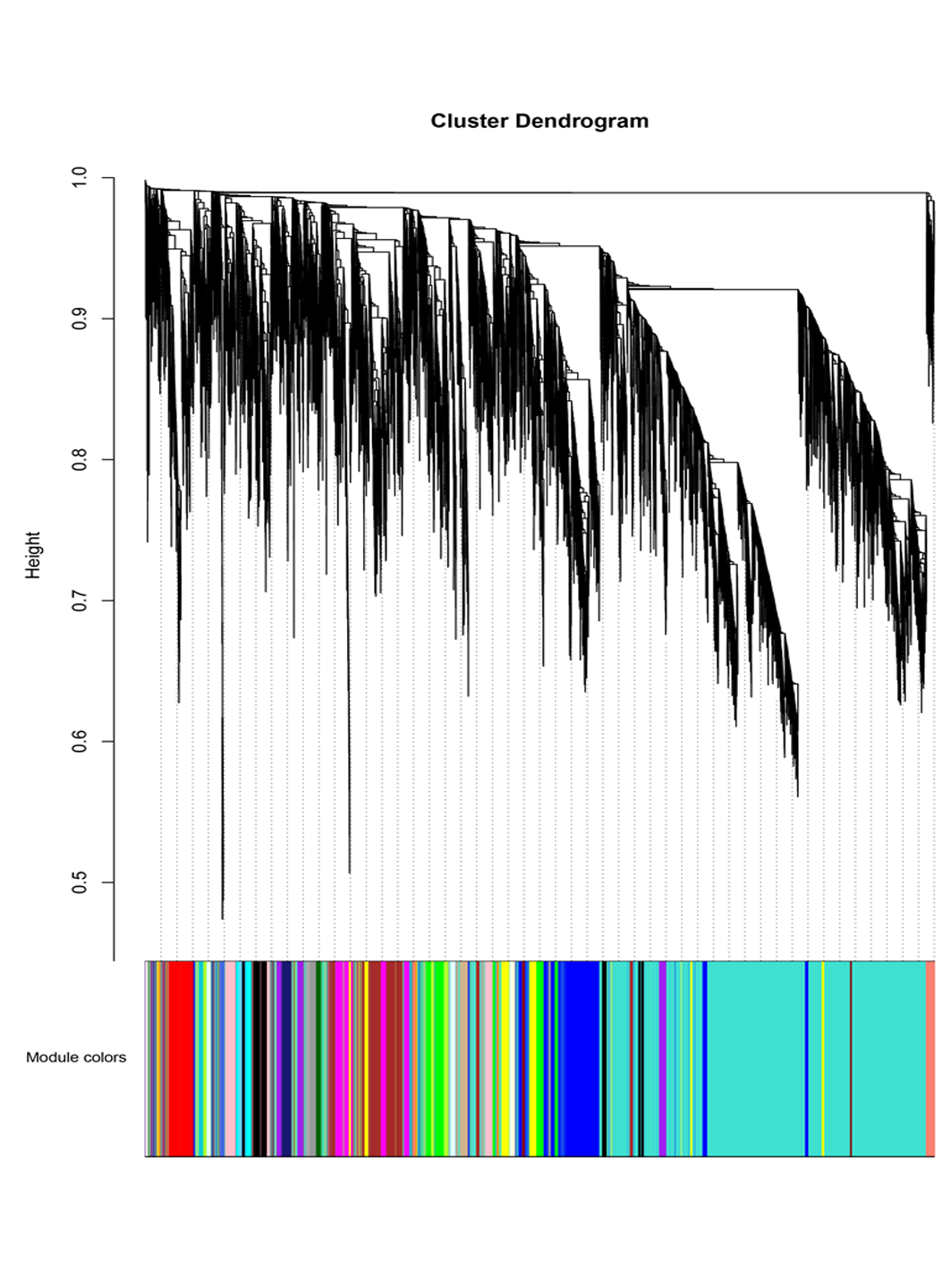

Supplement: Supplementary Figure 1 — Cluster dendrogram of the clustering of dissimilarity using a consensus topological overlap. Each module color represents one module. A total of 29 modules were identified. [file Image_1.TIF]

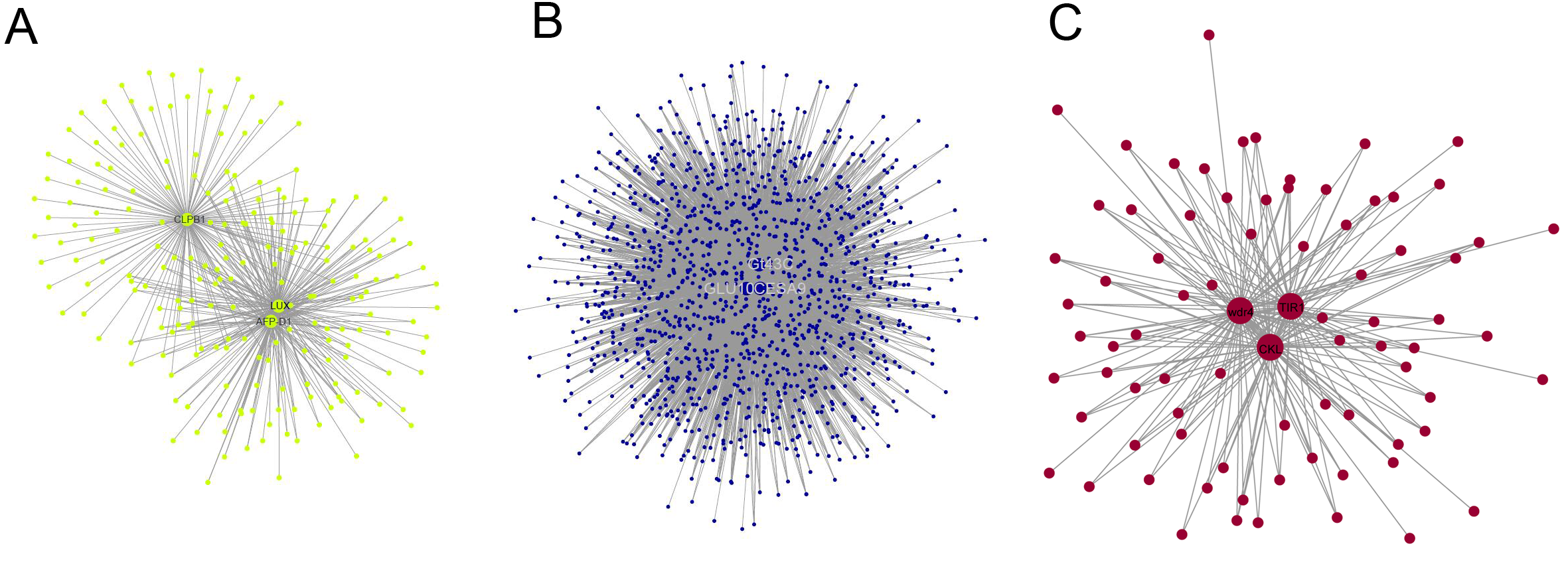

Supplement: Supplementary Figure 2 — Identification of hub gene in yellowgreen, blue and darkred modules by Cytoscape. (A) The hub gene in yellowgreen module. (B) The hub gene in blue module. (C) The hub gene in darkred module. [file Image_2.TIF]
